# The Role of PKR/eIF2α Signaling Pathway in Prognosis of Non-Small Cell Lung Cancer

**DOI:** 10.1371/journal.pone.0024855

**Published:** 2011-11-10

**Authors:** Yong He, Arlene M. Correa, Maria Gabriela Raso, Wayne L. Hofstetter, Bingliang Fang, Carmen Behrens, Jack A. Roth, Yihong Zhou, Liping Yu, Ignacio I. Wistuba, Stephen G. Swisher, Apar Pataer

**Affiliations:** 1 Department of Thoracic Surgery, Daping Hospital, Third Military Medical University, Chongqing, China; 2 Department of Thoracic and Cardiovascular Surgery, The University of Texas M.D. Anderson Cancer Center, Houston, Texas, United States of America; 3 Department of Pathology, The University of Texas M.D. Anderson Cancer Center, Houston, Texas, United States of America; 4 Department of Thoracic Head and Neck Medical Oncology, The University of Texas M.D. Anderson Cancer Center, Houston, Texas, United States of America; 5 Department of Neurological Surgery and Biological Chemistry, University of California Irvine, Irvine, California, United States of America; 6 Ziren Research LLC, Irvine, California, United States of America; University of Texas Southwestern Medical Center at Dallas, United States of America

## Abstract

**Background:**

In this study, we investigated whether PKR protein expression is correlated with mRNA levels and also evaluated molecular biomarkers that are associated with PKR, such as phosphorylated PKR (p-PKR) and phosphorylated eIF2α (p-eIF2α).

**Methodology and Findings:**

We determined the levels of PKR protein expression and mRNA in 36 fresh primary lung tumor tissues by using Western blot analysis and real-time reverse-transcriptase PCR (RT-PCR), respectively. We used tissue microarrays for immunohistochemical evaluation of the expression of p-PKR and p-eIF2α proteins. We demonstrated that PKR mRNA levels are significantly correlated with PKR protein levels (Spearman's rho = 0.55, *p*<0.001), suggesting that PKR protein levels in tumor samples are regulated by PKR mRNA. We also observed that the patients with high p-PKR or p-eIF2α expression had a significantly longer median survival than those with little or no p-PKR or p-eIF2α expression (*p* = 0.03 and *p* = 0.032, respectively). We further evaluated the prognostic effect of combined expression of p-PKR plus PKR and p-eIF2α plus PKR and found that both combinations were strong independent prognostic markers for overall patient survival on stage I and all stage patients.

**Conclusions:**

Our findings suggest that PKR protein expression may controlled by transcription level. Combined expression levels of PKR and p-PKR or p-eIF2α can be new markers for predicting the prognosis of patients with NSCLC.

## Introduction

The protein kinase (PKR) is an interferon-inducible serine/threonine kinase that mediates protein synthesis, a tightly regulated process that is critical in cellular proliferation and differentiation [1]–[3]. Increased PKR expression has been shown to correlate with better prognoses in head and neck cancer and colon cancer [2], [3], and accumulating evidence demonstrates that PKR may act as a tumor suppressor in leukemia and other hematopoietic malignancies [4], [5]. Binding of either double-stranded RNA (dsRNA) or structured single-stranded RNAs can mediate PKR phosphorylation [6], [7]. Activated PKR phosphorylates its well-documented downstream target, the alpha subunit of protein synthesis initiation factor eIF2 (eIF2α), leading to inhibition of protein synthesis and eliciting antiviral and antitumor activities [8], [9]. In addition to its role in translational control, PKR has been implicated in antiviral innate immunity, apoptosis, cell proliferation, and stress signaling [10]. Moreover, PKR expression and autophosphorylation are increased in several types of cancer, including melanoma, colon cancer, and breast cancer [11], [12]. The results of several studies have demonstrated the importance of phosphorylated eIF2α (p-eIF2α) in cancer therapy [10], [13], [14]: activation of the PKR-eIF2a phosphorylation pathway is essential for the antiproliferative and proapoptotic functions of the tumor suppressor gene [15].

Recently, we found that low expression of dsRNA-dependent PKR was significantly associated with shorter survival in NSCLC patients, suggesting that biologic functions of PKR or its downstream molecules could be valuable prognostic factors in NSCLC [1]. In this new study, our goal was to determine whether PKR protein expression is associated with its mRNA levels and whether its downstream targets, such as phosphorylated PKR (p-PKR) and p-eIF2a, are also prognostic factors in NSCLC. We first determined the PKR mRNA levels and protein expression in fresh-frozen NSCLC tissue and found a positive correlation between PKR protein expression and mRNA levels. Next, using immunohistochemical staining, we investigated the expression of p-PKR and its well-characterized downstream molecule p-eIF2α in archived tissue microarray specimens. Our results show that p-PKR and p-eIF2α are predictive biomarkers of NSCLC outcomes and that when expression of PKR was combined with expression of p-PKR or p-eIF2α, the effect on predicting patient survival was enhanced.

## Results

### PKR protein expression correlates with mRNA levels

**Figure 1:**
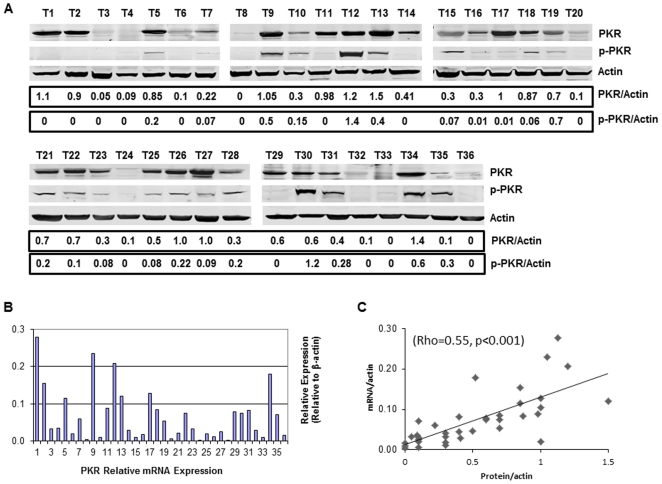
PKR mRNA levels are correlated with PKR protein levels in primary NSCLC tissues. **A.** Western blot analysis of PKR and p-PKR protein in tumor samples. A densitometric analysis of the ratio of PKR or PKR to β-actin is represents normalized protein levels. Each lane represents a tumor sample from an individual patient. **B.** mRNA levels of corresponding samples were determined using quantitative real-time PCR. Levels of β-actin protein and its mRNA of the same sample were used as controls. **C.** Scatter plot of PKR protein expression correlated with mRNA expression (Spearman's rho = 0.55, *p*<0.001).

To investigate whether PKR protein expression is associated with mRNA levels, we determined the PKR and p-PKR protein expression and PKR mRNA levels in 36 fresh primary lung tumor tissues using Western blot analysis and real time RT-PCR, respectively. Protein expression of β-actin and its mRNA levels were also determined and used as controls. The results of the Western blotting analyses showed that tumor samples expressed different levels of PKR at both protein and mRNA levels (Figure 1A and 1B). Statistical analysis revealed a significant correlation between PKR protein expression and its mRNA levels. (Spearman's rho = 0.55, *p*<0.001; Figure 1C). These results suggest that PKR gene expression may cause of the differing levels of PKR protein expression in tumors.

### Correlation between p-PKR and p-eIF2α protein expression in NSCLC tumors with clinicopathologic features

**Figure 2:**
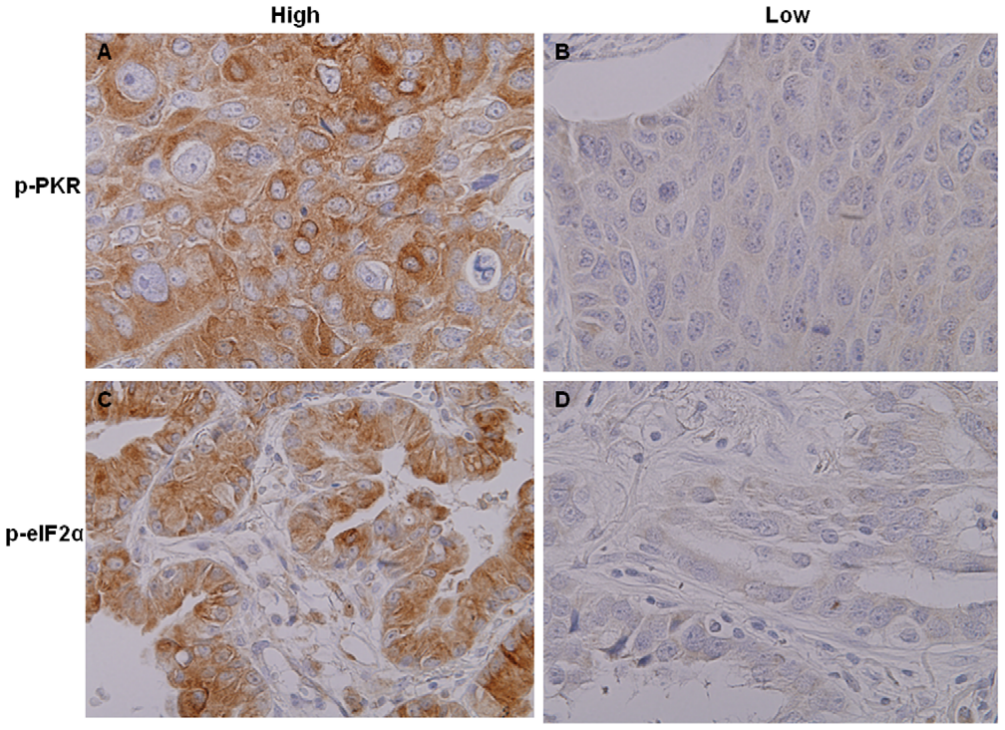
Representative results of immunohistochemical staining of NSCLC tumor specimens for p-PKR and p-eIF2α. High-expressing cases (**A** and **C**). Low-expressing cases (**B** and **D**). Expression of p-PKR and p-eIF2α was detected in the cytoplasm.

Because the biological function of PKR is closely associated with its phosphorylation, and eIF2α phosphorylation is a hallmark of PKR activation, we next evaluated the expression of p-PKR and p-eIF2α proteins in TMA specimens using immunohistochemical staining. Table 1 summarizes the relationships between p-PKR or p-eIF2α expression and other clinicopathologic features. High p-PKR expression was associated with the adenocarcinomas subtype (*p*<0.001). No correlation was observed between p-PKR expression and gender sex, TNM stage, or smoking status. We also found no correlation between p-eIF2α expression and clinicopathologic features. Representative images of immunohistochemical staining results for p-PKR and p-eIF2α are shown in Figure 2. The p-PKR and p-eIF2α proteins were expressed in the cytoplasm of tumor cells.

**Table 1:** Relationships between the level of p-PKR and p-eIF2α expression and clinicopathologic characteristics in TMA of NSCLC patients.

Characteristics	p-PKR Score		*p*-Value	p-eIF2α Score		*p*-Value
	Low (< = 70)	High (>70)		Low (< = 150)	High (>150)	
Gender			0.11			0.45
Male	49 (50.0)	34 (37.6)		39 (43.3)	44 (49.4)	
Female	49 (50.0)	56 (62.4)		51 (56.7)	45 (50.6)	
Pathological TNM			0.33^a^			0.25^a^
Stage I	62 (62.9)	58 (64.4)		55 (60.0)	57 (64.8)	
Stag II	19 (19.6)	13 (14.4)		17 (18.9)	16 (18.2)	
Stage III–IV	17 (17.5)	19 (21.2)		19 (21.1)	15 (17.0)	
pT			0.11^b^			0.46^b^
T1	26 (26.5)	35 (38.9)		27 (30.1)	30 (33.7)	
T2	64 (65.3)	47 (52.2)		56 (62.2)	48 (53.9)	
T3	3 (3.1)	5 (5.6)		3 (3.3)	6 (6.8)	
T4	5 (5.1)	3 (3.3)		4 (4.4)	5 (5.6)	
pN			0.48^c^			0.06^c^
N0	67 (68.4)	62 (68.9)		57 (63.3)	67 (75.3)	
N1	20 (20.4)	13 (15.4)		19 (21.1)	14 (15.7)	
N2	11 (11.2)	14 (15.7)		14 (15.6)	8 (9.0)	
pM			0.07			0.36
M0	96 (97.9)	84 (93.3)		86 (95.5)	86 (96.6)	
M1	2 (2.1)	6 (6.7)		4 (4.4)	3 (3.4)	
Histologic type			<0.01			0.19
ACC	41 (41.4)	73 (82.0)		51 (56.7)	56 (62.5)	
SCC	58 (58.6)	16 (18.0)		39 (43.3)	33 (37.5)	
Tobacco history			0.11			0.13
No	25 (25.5)	33 (36.3)		29 (32.2)	19 (21.8)	
Yes	72 (74.5)	58 (63.7)		61 (67.8)	68 (78.2)	

a The *p*-value was calculated between pathologic stage I and II–IV.

b Between T1 and T2–T4.

c Between N0 and N1–N2.

SCC, squamous cell carcinoma. ACC, Adenocarcinoma.

### Correlation between p-PKR and p-eIF2α protein expression in NSCLC tumors with disease outcomes

**Figure 3:**
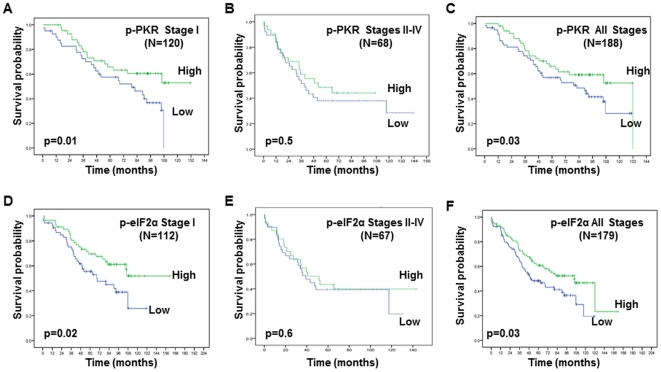
The prognostic significance assessed by using Kaplan-Meier survival estimates and long-rank test. **A–F.** Kaplan-Meier survival curves according to the differences in (**A**, **B**, **C**) p-PKR and (**D**, **E**, **F**) p-eIF2-α expression in patients with stage I (**A**, **D**), stages II–IV (**B**, **E**) and all stage (**C**, **F**) NSCLC. The survival rates were significantly worse in the patients with low p-PKR or p-eIF2α expression than in those with high p-PKR or p-eIF2α expression (Stage I: *p* = 0.01 and *p* = 0.02; All stages: *p* = 0.03 and *p* = 0.03, respectively).

To further evaluate whether p-PKR or p-eIF2α protein expression correlates with clinical outcomes of patients with NSCLC, Kaplan–Meier analysis revealed that for stage I and all stages, patients with relatively elevated p-PKR had significantly longer survival than those with little or no p-PKR expression (Figure 3A and 3C). For all stage patients, the median survival time was 105 months for patients with high p-PKR expression and 51 months for those with little or no p-PKR expression. A significant association was also observed between high p-eIF2α protein expression and longer survival on stage I and all stages (Figure 3D and 3F). There is no significant association was observed between high p-PKR or high p-eIF2α protein expression and longer survival on stages II–IV (Figure 3B and 3E).

In a univariate analysis, a Cox proportional hazards model indicated that p-PKR and p-eIF2α had prognostic significance (Table 2). In a multivariate analysis, we found that p-PKR and p-eIF2α expression were statistically significant associated with survival (Table 2). These results indicate that p-PKR and p-eIF2α expression are independent biomarkers of patients' survival.

**Table 2:** Univariate and multivariate Cox model assessing effects of covariates on overall survival.

	Stage I		All Stages	
Characteristics	Hazard ratio (95% CI)	*p*-Value	Hazard ratio (95% CI)	*p*-Value
A. Univariate Cox regression model				
Age	0.99 (0.99–1.00)	0.21	0.99 (0.96–1.02)	0.48
Gender (male vs female)	1.49 (0.88–2.51)	0.14	1.31 (0.87–1.96)	0.19
Tobacco history (yes vs no)	1.50 (0.78–2.89)	0.22	0.85 (0.54–1.33)	0.48
Pathological TNM stage				
Stage II+III+IV vs I	-	-	2.79 (1.65–4.71)	0.001
Histologic type (ACC vs SCC)	1.36 (0.67–2.78)	0.39	2.00 (0.58–6.85)	0.27
p-PKR (High vs Low)	0.45 (0.24–0.84)	0.01	0.51 (0.28–0.96)	0.02
p-eIF2a (High vs Low)	0.53 (0.31–0.92)	0.03	0.54 (0.32–0.96)	0.03
B. Multivariate Cox regression model				
Pathological TNM stage				
Stage II+III+IV vs I	-	-	2.71 (1.53–4.82)	0.001
p-PKR (High vs Low)	-	-	0.56 (0.34–0.95)	0.03
p-eIF2a (High vs Low)	-	-	0.61 (0.42–0.97)	0.04

**Figure 4:**
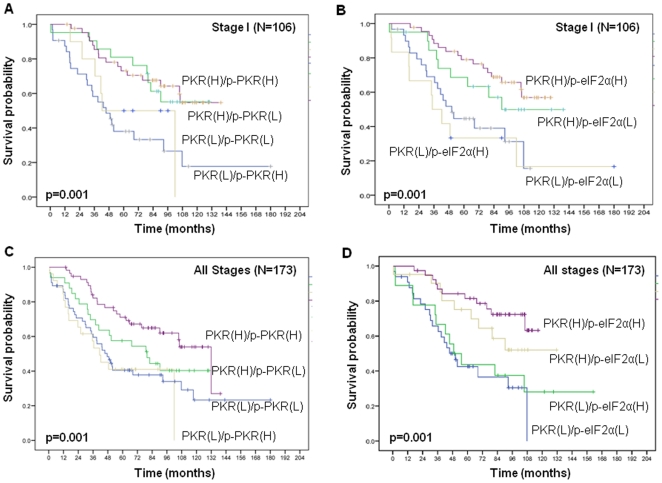
The prognostic significance assessed by using Kaplan-Meier survival estimates and long-rank test. **A** and **C**. Survival rate was significantly lower patients with low PKR or low p-PKR expression than in those with high PKR or high p-PKR expression on stage I (A) and all stages (C) (*p* = 0.001 and *p* = 0.001, respectively). **B** and **D**. Survival rate was also significantly lower in patients with low PKR or low p-eIF2α expression than in those with high PKR or high p-eIF2α expression on stage I (B) and all stages (D) (*p* = 0.001 and *p* = 0.001, respectively).

We also examined the associations between the expression levels of PKR, p-PKR, and p-eIF2α. Our results indicated that p-PKR expression significantly correlated with p-eIF2a expression (Spearman's rho = 0.48, *p*<0.001). PKR expression also significantly correlated with expression of p-PKR (Spearman's rho = 0.31, *p* = 0.004) and p-eIF2α (Spearman's rho = 0.45, *p*<0.001). We further evaluated the prognostic effect of combined expression of p-PKR plus PKR and p-eIF2α plus PKR and found that both combinations were strong independent prognostic markers for overall patient survival on stage I (Figure 4A and 4B) and all stage patients (Figure 4C and 4D). For all stage patients, patients with high PKR and high p-PKR expression had a median survival time of 132 months, which was significantly longer than that of patients with high PKR and low p-PKR expression (median survival, 80 months; Figure 4C). Patients with little or no PKR and p-PKR expression had a significantly shorter median survival (43 months; Figure 4C). There is a difference in outcome for PKR(H)/p-PKR(H) vs PKR(L)/p-PKR(H) patients on Figure 4A. Our IHC score range is 0–300 for PKR and p-PKR. In current study, we are using median cut-off (150 for PKR and 70 for p-PKR). On Figure 4A, 106 stage I patients divided into four groups, 43 patients with high PKR and high p-PKR, 21 patients with high PKR and low p-PKR expression, 32 Patients with low PKR and p-PKR expression and 10 patients with low PKR and high p-PKR expression. These 10 patients have p-PKR score range 80–120, are very close to median cut-off score (70), and may also belong to low PKR low p-PKR group. For all stage patients, patients with high PKR and high p-eIF2α expression had a median survival time of 132 months, which was significantly longer than that of patients with high PKR plus low p-eIF2α expression (median survival, 80 months) and those with low PKR plus high p-eIF2α expression (median survival, 47 months; Figure 4D). Finally, patients with little or no expression of both PKR and p-eIF2α had a significantly short median survival (median survival, 35 months; Figure 4D).

## Discussion

The results of this study show that PKR mRNA levels are associated with PKR protein expression in primary NSCLC tumors. Our findings suggest that PKR protein expression may control transcription levels. Further, our data suggest that real time RT-PCR, a sensitive method for quantitatively analyzing mRNA levels, can be used to determine mRNA levels in biopsy samples and might therefore be useful for predicting patient survival. We also found that patients with high p-PKR expression had significantly longer median survival than those with little or no p-PKR protein expression. Results of various studies of human malignancies have suggested that high PKR expression indicates favorable prognosis for patients with squamous cell carcinoma of the head or liver [2], [16], thus suggesting that PKR may play an important role in suppressing tumor progression and affecting apoptosis [17]. We have also studied PKR pathways and have found them to be clearly necessary for inducing apoptosis in some cancer cells, including lung cancer, after certain treatments such as melanoma differentiation-associated gene 7 (*MDA7*), E2F-1, and TNFα [18], [19]. More recently, we and others demonstrated that some small compounds can induce PKR-dependent apoptosis in both cancer cells and murine embryonic fibroblasts [20], [21], indicating that modulating PKR activity could be an interesting approach to cancer therapy. Our finding that NSCLC tumor cells that express a high level of p-PKR correlate with a favorable prognosis is consistent with previous observations that PKR activation is associated with apoptosis induction.

Our findings also demonstrated that patients with high expression of both PKR and p-PKR had significantly longer survival than did those with other combinations of expression levels, including those positive for PKR and negative for p-PKR and those negative for both PKR and p-PKR. Our observation that 53% of the NSCLC tumor samples highly expressed PKR and that 61% of them highly expressed p-PKR [data not shown] These results led us to speculate that PKR expression (i.e., PKR) and PKR activation (i.e., p-PKR) are affected by differing expression of the PKR activator or PKR inhibitor. Other investigators have reported that the function of PKR can be regulated by cellular proteins either positively (e.g., MDA-7/interleukin-24 and PKR-activating protein [PACT]) or negatively (e.g., p58IPK, nucleophosmin and heat shock proteins 90 and 70) [18], [19]. In future studies, we will seek to determine which PKR activators and inhibitors affect the PKR signaling pathway in NSCLC tumor samples.

We observed that tumors with high p-eIF2α expression had a significantly longer median survival. In additional, we demonstrated that patients with high expressions of both PKR and p-eIF2α also had significantly longer survival than did those with other combinations of expression levels. Our finding that 47% of the tumor samples had low PKR expression and 27% had high p-eIF2α expression [data not shown]. These results suggest that eIF2α phosphorylation in some NSCLC tumors occurs independent of the PKR pathway. Besides PKR, three different eIF2α kinases that can phosphorylate eIF2α in response to various stress conditions have been identified: heme-regulated inhibitor, the homologue of *Saccharomyces cerevisiae* protein kinase general control non-derepressible-2, and RNA-dependent-protein-kinase-like endoplasmic reticulum (ER) kinase (PERK; also known as pancreatic eIF2α kinase) [13].

In conclusion, our data suggest that the PKR/phosphorylated PKR/phosphorylated eIF2α signaling pathway plays an important role in the prognosis for non-small cell lung cancer (NSCLC). PKR pathway activities may be useful for predicting NSCLC outcomes, and modulating PKR pathway activities might be a potential NSCLC treatment option.

## Methods

### Patients and Tissues

NSCLC patients who were undergoing radical resection of their primary cancer at The University of Texas M. D. Anderson Cancer Center between 2007 and 2008 were used for this study based on availability. Patients were excluded from the study if they had previously undergone radiotherapy or chemotherapy for cancer. Patients all provided written informed consent for the use of their tissues, and the study was approved by our Institutional Review Board (University of Texas, MD Anderson Cancer Center). After being surgically resected, each fresh tumor was immediately divided into two portions; one was instantly frozen and stored in liquid nitrogen for protein and RNA extraction, and the other was fixed with formalin and embedded in paraffin for routine histopathologic evaluation and diagnosis. Tissues with an estimated tumor cell content of 70% or more were used for molecular analyses. In addition to our patients' tissues, we obtained 193 NSCLC Tissue microarray (TMA) specimens (114 adenocarcinomas, and 74 squamous cell carcinomas) between 1997 and 2001 from the Lung Cancer Specialized Program of Research Excellence Tissue Bank at M. D. Anderson Cancer Center (Houston, TX). All specimens were histologically examined and classified using the 2004 World Health Organization classification system [22]. In most cases, detailed clinical and pathologic information, including the patients' demographic data, smoking history, and overall survival plus the disease TNM staging and time to recurrence, was available (Table 1).

### Western Blot Analysis

Frozen tumor tissues were initially preparared by being washed twice in cold PBS. Approximately 20 mg of tissue from each fresh sample was homogenized in 0.5 ml ice-cold lysis buffer (1% NP40, 50 mM HEPES [pH 7.4], 150 mM NaCl, 1.5 mM MgCl_2_, 100 mM NaF, 1 mM EGTA, 1 mM Na_3_VO_4_, 10% glycerol, and 10 mM Na pyrophosphate [Roche Applied Science]), containing freshly added protease and phosphate inhibitor. The lysates were spun at 14,000 g in a microcentrifuge at 4°C for 10 min, and the resulting supernatants were used as tissue extracts. The extracts, equivalent to 60 µg of the total protein, were separated by using a 10% SDS-polyacrylamide gel and then transferred to nitrocellulose membranes. The membranes were blocked with TBS containing 5% nonfat dried milk and then probed in PBS containing 5% bovine serum. The following antibodies were used: rabbit anti-PKR (K-17; Santa Cruz Biotechnology), rabbit anti-p-PKR (pT446) (Epitomics), rabbit anti-p-eIF2a (Epitomics), and mouse anti-β-actin (Sigma). Immunoreactive bands were detected and quantified using a Li-Cor Odyssey infrared imaging system.

### Reverse Transcription and Real-time Absolute Quantitative Reverse-Transcription-Polymerase Chain Reaction (Real-Time AqRT-PCR)

The total RNA (1 µg) from each frozen clinical sample was extracted using a Trizol extraction kit (Invitrogen) and reverse transcribed in a 20 µL reaction volume by using Taqman reverse-transcription reagents (Applied Biosystems) according to the manufacturer's instructions. The cDNAs were diluted and quantified for expression PKR using real-time RT-PCR (SYBR Green I) (performed by Ziren Research LLC, Irvine, CA). A single standard was incorporated to determine the absolute ratio of expression of each target and reference gene, as previously described [23]. The primer sequences for PKR were as follows: forward, 5′-TCTTCATGTATGTGACACTGC-3′, and reverse, 5′-CACACAGTCAAGGTCCTT AG-3′.

### Immunohistochemical Staining and Evaluation

The antibodies used for Western blot analysis were also used for immunohistochemical staining. Formalin-fixed and paraffin-embedded tissue histology sections (5-µm thick) were deparaffinized, hydrated and heated in a steamer for 10 min with 10 mmol/L of sodium citrate (pH 6.0) for antigen retrieval. Peroxidase was blocked with 3% H_2_O_2_ in methanol at room temperature for 15 min, followed by incubation in 10% bovine serum albumin in TBS-t for 30 min. The slides were next incubated with primary antibody at 1∶100 dilutions for 65 min at room temperature. After being washed with PBS, the slides were incubated with biotin-labeled secondary antibody for 30 min. Finally, the samples were incubated with streptavidin-peroxidase at a 1∶40 dilution for 30 min. The samples were then stained with 0.05% 3′, 3-diaminobenzidine tetrahydrochloride prepared in 0.05 mol/L of Tris buffer (pH 7.6) containing 0.02% H_2_O_2_ and subsequently counterstained with hematoxylin. As a positive control, formalin-fixed and paraffin-embedded lung tissues with normal bronchial epithelia were used. As a negative control, tissue samples not incubated with the primary antibody were used. Immunohistochemical staining was quantified by two independent pathologists (Drs. Raso and Pataer) with a four-value intensity score described previously [1].

### Statistical Analysis

The median was used as the cutoff point for p-PKR and p-eIF2a. The biomarkers were dichotomized into low- and high-level groups as follows: p-PKR: low (score≤70), high (score>70); and p-eIF2a: low (score≤150), high (score>150). In univariate analysis, independent sample *t* and *X^2^* tests were used to analyze continuous and categorical variables, respectively. Survival probability as a function of time was computed by using the Kaplan-Meier estimator. The log-rank test was used for between-group comparisons of patient survival time. The Cox proportional hazards model was used to calculate the influence of p-PKR and p-eIF2a expression on survival time, with adjustments made for clinical and histopathologic parameters (age, sex, smoking status and tumor histologic subgroup. The two-sided test was used to test equal proportion between groups in two-way contingency tables. The generalized estimating equation approach was used to estimate differences in the means for the data. Statistical significance was set at P<0.05.
